# The Human Fetal and Adult Stem Cell Secretome Can Exert Cardioprotective Paracrine Effects against Cardiotoxicity and Oxidative Stress from Cancer Treatment

**DOI:** 10.3390/cancers13153729

**Published:** 2021-07-24

**Authors:** Federico Villa, Silvia Bruno, Ambra Costa, Mingchuan Li, Michele Russo, James Cimino, Paola Altieri, Clarissa Ruggeri, Cansu Gorgun, Pierangela De Biasio, Dario Paladini, Domenico Coviello, Rodolfo Quarto, Pietro Ameri, Alessandra Ghigo, Silvia Ravera, Roberta Tasso, Sveva Bollini

**Affiliations:** 1 Cellular Oncology Unit, IRCCS Ospedale Policlinico San Martino, 16132 Genova, Italy; federico.villa@hsanmartino.it (F.V.); cansu.gorgun@edu.unige.it (C.G.); rodolfo.quarto@unige.it (R.Q.); 2 Department of Experimental Medicine (DIMES), University of Genova, 16132 Genova, Italy; silvia.bruno@unige.it (S.B.); ambra.costa@edu.unige.it (A.C.); silvia.ravera@unige.it (S.R.); 3 Department of Molecular Biotechnology and Health Sciences, University of Torino, 10126 Torino, Italy; mingchuanli@jnu.edu.cn (M.L.); m.russo@unito.it (M.R.); james.cimino@unito.it (J.C.); alessandra.ghigo@unito.it (A.G.); 4 Laboratory of Cardiovascular Biology, Department of Internal Medicine (DIMI), University of Genova, 16132 Genova, Italy; paola.altieri@unige.it (P.A.); clarissa.ruggeri@edu.unige.it (C.R.); pietroameri@unige.it (P.A.); 5 Unit of Prenatal Diagnosis and Perinatal Medicine, IRCCS Ospedale Policlinico San Martino, 16132 Genova, Italy; pierangela.debiasio@hsanmartino.it; 6 Fetal Medicine and Surgery Unit, IRCCS Istituto Giannina Gaslini, 16147 Genova, Italy; Dariopaladini@gaslini.org; 7 Human Genetics Laboratory, IRCCS Istituto Giannina Gaslini, 16147 Genova, Italy; domenicocoviello@gaslini.org; 8 Cardiovascular Disease Unit, IRCCS Ospedale Policlinico San Martino, 16132 Genova, Italy

**Keywords:** stem cell, paracrine effect, cardiomyocyte, doxorubicin, cardiotoxicity, mitochondria, oxidative stress, cancer treatment

## Abstract

**Simple Summary:**

Anthracyclines, such as doxorubicin (Dox), are an important class of chemotherapeutic drugs. However, their use is hampered by the risk of developing heart failure. The aim of this study was to assess and compare the cardioprotective effects exerted by a set of factors, collectively named secretomes, secreted by either adult or fetal human stem cells. Both secretome formulations were effective in counteracting Dox-induced apoptosis and mitochondrial impairment in cardiomyocytes and cardiac fibroblasts. In vivo experiments in a mouse model of Dox-induced cardiomyopathy (DIC) indicated that early administration of both secretomes during Dox treatment exerted beneficial long-term effects, preserving cardiac function and body mass. These findings suggest that the stem cell secretome could represent a feasible option for future paracrine cardioprotective therapy against Dox-related cardiotoxicity during cancer treatment.

**Abstract:**

Cardiovascular side effects are major shortcomings of cancer treatments causing cardiotoxicity and late-onset cardiomyopathy. While doxorubicin (Dox) has been reported as an effective chemotherapy agent, unspecific impairment in cardiomyocyte mitochondria activity has been documented. We demonstrated that the human fetal amniotic fluid-stem cell (hAFS) secretome, namely the secreted paracrine factors within the hAFS-conditioned medium (hAFS-CM), exerts pro-survival effects on Dox-exposed cardiomyocytes. Here, we provide a detailed comparison of the cardioprotective potential of hAFS-CM over the secretome of mesenchymal stromal cells from adipose tissue (hMSC-CM). hAFS and hMSC were preconditioned under hypoxia to enrich their secretome. The cardioprotective effects of hAFS/hMSC-CM were evaluated on murine neonatal ventricular cardiomyocytes (mNVCM) and on their fibroblast counterpart (mNVFib), and their long-term paracrine effects were investigated in a mouse model of Dox-induced cardiomyopathy. Both secretomes significantly contributed to preserving mitochondrial metabolism within Dox-injured cardiac cells. hAFS-CM and hMSC-CM inhibited body weight loss, improved myocardial function, reduced lipid peroxidation and counteracted the impairment of mitochondrial complex I activity, oxygen consumption, and ATP synthesis induced by Dox. The hAFS and hMSC secretomes can be exploited for inhibiting cardiotoxic detrimental side effects of Dox during cancer therapy, thus ensuring cardioprotection via combinatorial paracrine therapy in association with standard oncological treatments.

## 1. Introduction

Good communication and networking capacities are mandatory skills to ensure efficient performance. Stem cells and progenitors have been broadly demonstrated to master such activity by means of their paracrine potential. Although progenitor cells were initially believed to have the potential to develop into new viable tissue, almost two decades of translational research have shown that stem cell transplantation resulted in meager engraftment and poor survival in the host, with a lack of functional maturation into tissue-specific cells. Nevertheless, several independent preclinical studies have provided evidence that the transplanted cells can release trophic paracrine factors which activate endogenous pathways, leading to the significant improvement of tissue repair [1]. In this perspective, the stem/progenitor cell *secretome*, i.e., the whole of the cell-secreted paracrine factors, including both soluble individual molecules and membrane-bound extracellular vesicles, has gained increasing interest as a novel curative booster in regenerative medicine [2]. Therefore, its role is being increasingly scrutinized as a unique therapeutic cell-free strategy with appealing potential in different clinical applications, including cardiovascular disorders [3,4]. As a matter of fact, multiple studies have suggested harnessing the stem/progenitor cell secretome as a therapeutic agent for cardiac disease and myocardial dysfunction and have provided proof of principle of the paracrine therapy [5,6,7]. Recent evidence advocates the importance of the paracrine mechanism of stem cell action in enhancing endogenous cardiac reparative pathways, following their delivery into the injured heart. A large amount of data highlights the relevance of conditioning the tissue microenvironment where stem cell-released factors induce cardiomyocyte survival, modulate inflammation, and support local angiogenesis in a temporal and spatial manner [8]. Within this scenario, one of the main attractive paracrine phenomena of the mesenchymal stromal cell secretome is represented by the cardioprotective effect underpinning reduced cardiomyocyte apoptosis and improving cardiac function in the compromised myocardium [9,10].

In recent years, cardiovascular complications of oncological therapies have emerged as a critical aspect affecting the life quality and expectancy of cancer survivors [11,12]. Indeed, the field of cardio-oncology has quickly developed to address and counteract cardiotoxicity and cardiac dysfunction as the major drawback of oncological drugs and cancer treatments. Anthracyclines, including doxorubicin (Dox), are well-known pharmacological agents broadly prescribed to treat a variety of solid tumors and hematologic malignancies; nonetheless, their effective clinical applications are burdened by cardiotoxic side effects that may result in progressive cardiac remodeling, contractile impairment, cardiomyopathy, and heart failure [13,14,15]. Although Dox cardiotoxicity is diagnosed when clinically evident or when the left ventricular (LV) ejection fraction decreases below a certain threshold, thus, marginally capturing the cellular and molecular events caused by Dox in the heart [16]. Comprehensive biochemical studies have recognized various mechanisms of Dox-induced damage on cardiac cells, including alteration of redox homeostasis with the generation of reactive oxygen species (ROS) and blockage of topoisomerase II-β, both of which can lead to DNA damage and premature cardiomyocyte senescence and apoptosis [17,18,19].

Consistent with the fact that clinical manifestations represent only a minor part of the spectrum of Dox cardiotoxicity, surveillance programs for early recognition of Dox side effects have been proposed and partially validated, but prevention strategies are still lacking. Recent evidence points towards the Dox-related deregulation of mitochondrial function and structural organization as a crucial aspect underlying anthracycline-induced cardiotoxicity. Therefore, therapeutic strategies aiming to preserve mitochondrial function and metabolism are receiving increasing consideration to prevent/delay cardiotoxic injury [20].

In recent years, the scientific community has also focused on exploiting stem cell modulatory effects as a pioneering strategy to provide cardioprotection against anthracycline-induced cardiotoxicity and cardiomyopathy. Paracrine conditioning of the cardiac microenvironment may offer an appealing strategy as a therapeutic combinatorial approach in association with standard oncological treatment. Recently, our group demonstrated that the soluble paracrine factors released by human fetal mesenchymal stromal cells isolated from leftover samples of II trimester amniotic fluid—namely amniotic fluid-derived stem cells (hAFS)—can efficiently antagonize senescence and apoptosis of cardiomyocytes and cardiac progenitor cells, two major features of anthracycline-derived cardiotoxicity. Notably, the hAFS-conditioned medium inhibited Dox-elicited senescence and apoptosis in murine cardiomyocytes in vitro, with decreased DNA damage, and upregulation of the NF-kB controlled genes, *Il6* and *Cxcl1*, thus promoting their survival [21].

To further progress research in this field, we provided a detailed study as proof of principle for stem cell-based paracrine therapy of chemotherapy-related cardiotoxicity. In particular, we aimed to address two main aspects: (i) comparison of the therapeutic potential of the secretomes from human fetal stem cells and adult somatic mesenchymal stromal progenitors in a preclinical model of Dox-induced cardiac injury, and (ii) analysis of their cardioprotective influence—in terms of mitochondria structure and metabolism—in injured cardiomyocytes.

## 2. Materials and Methods

### 2.1. Cell Culture

Human amniotic fluid-derived stem cells (hAFS) were isolated from leftover samples of II trimester human amniotic fluid during routine prenatal screening via amniocentesis. Samples were obtained from the Prenatal Diagnosis and Perinatal Medicine Unit, IRCCS San Martino Hospital, the Fetal and Perinatal Medical and Surgery Unit, and Human Genetics Laboratory at the IRCCS Istituto Gaslini Hospital (Genova, Italy) after collecting informed written consent from all donors and in compliance with local ethical committee authorization (protocol P.R. 428REG2015) and with Helsinki Declaration guidelines. hAFS were isolated based on c-KIT expression via immunomagnetic sorting (CD117 MicroBead Kit, Miltenyi Biotec, Bologna, Italy) and cultured in Minimal Essential Medium (MEM)-alpha with 15% FBS (Gibco–Thermo Fisher Scientific, Monza, Italy), 18% Chang B and 2% Chang C Medium (Irvine Scientific, Santa Ana, CA, USA) with 1% L-glutamine and 1% penicillin/streptomycin (Gibco–Thermo Fisher Scientific, Monza, Italy), in an incubator at 37 °C with 5% CO_2_ and 20% O_2_ atmosphere, as previously described [21,22,23]. Human adipose tissue-derived mesenchymal stromal cells (hMSC) were obtained from subcutaneous adipose tissue in compliance with Regione Liguria Ethical Committee authorization (P.R. 23571). Liposuction aspirates obtained from human healthy donors were rinsed with cold phosphate-buffered saline (PBS) and digested in 0.1% type I collagenase (Gibco—Thermo Fisher Scientific, Monza, Italy) in PBS at 37 °C for 60 min. Following this, the stromal vascular fraction (SVF) pellet was rinsed and cultured in Dulbecco’s MEM (D-MEM, Biochrom GmbH, Berlin, Germany) supplemented with 10% fetal bovine serum (FBS), 2 mM of l-glutamine, and 50 mg/mL penicillin/streptomycin (all Gibco–Thermo Fisher Scientific, Monza, Italy). The cultures were performed in the presence of 1 ng/mL of fibroblast growth factor-2 (FGF-2; Peprotech, London, UK), as previously described [24].

Primary cultures of mouse neonatal ventricular cardiomyocytes (mNVCM) and neonatal mouse cardiac fibroblasts (mNVFib) were obtained from wild type C57Bl6/J 2-days-old mouse pups in compliance with national and European international standards of animal care and according to the required authorization from the Italian Ministry of Health (authorization n. 384/2016-PR and n. 792/2015-PR from the Italian Ministry of Health). mNVCM were obtained via enzymatic digestion of the cardiac tissue by papain with thermolysin enzymes (Pierce Primary Cardiomyocyte Isolation Kit, Thermo Fisher Scientific, Monza, Italy), mNVCM were seeded on 0.2% gelatin (Sigma-Aldrich, Merck, Darmstadt, Germany) and fibronectin (Sigma-Aldrich, Merck, Darmstadt, Germany, 1:1000 diluted solution) coated wells as 10^5^ cells/cm^2^ in complete medium (Dulbecco’s Modified Eagle Medium, DMEM, with 10% heat-inactivated FBS and 1% penicillin/streptomycin, all Gibco–Thermo Fisher Scientific, Monza, Italy), in an incubator at 37 °C with 5% CO_2_ and a 20% O_2_ atmosphere. mNVFib were isolated by treating the cardiac tissue with multiple digestions in a collagenase II solution (300 U/mL, Worthington Biochemicals) as the first adherent stromal population during the pre-plating step, according to [25]. mNVFib were then cultured at 2 × 10^3^ cells/cm^2^ in complete medium in an incubator at 37 °C with 5% CO_2_ and 20% O_2_ atmosphere.

The Dox-sensitive 4T1 breast cancer cell line was purchased from the American Type Culture Collection (ATCC, Manassas, VA, USA) and cells were cultured in vitro as previously reported [19].

### 2.2. Collection of the hAFS- and the hMSC-Conditioned Medium

In order to boost the release of cardio-protective paracrine factors, hAFS and hMSC were primed in vitro for 24 h in serum-free medium under 1% O_2_, as previously optimized [21,23,24]. The hAFS and hMSC secretome, namely the total amount of soluble factors cells secreted in their conditioned medium (hAFS-CM and hMSC-CM, respectively), was collected following hypoxic preconditioning and concentrated using ultrafiltration membranes with a 3kDa selective cut-off (Amicon Ultra-15, Millipore, Burlington, MA, USA). Protein concentration was evaluated by means of the BiCinchoninic Acid (BCA) assay (Thermo Fisher Scientific, Monza, Italy). hAFS-CM and hMSC-CM samples were then stored at −80 °C and used in experiments as 40 μg/mL of solution from a previous protocol [21].

### 2.3. In Vitro Experimental Outline

mNVCM, mNVFib, and 4T1 cells were incubated in low-serum complete medium (0.5% heat-inactivated FBS-supplemented medium) for 1 h, washed in PBS solution, and then treated with either hAFS-CM or hMSC-CM for 3 h in serum-free conditions (serum-free medium: DMEM medium with 1% penicillin/streptomycin, all from Gibco–Thermo Fisher Scientific, Monza, Italy) before being exposed to 1 µM Dox (from a 2 mg/mL solution courtesy of Dr. Sabrina Beltramini, Director of the Antiblastic Drug Unit, IRCCS AOU San Martino-IST, Genova, Italy). PI3K inhibitors (all from MedChemExpress, NJ, USA) were added to mNVCM together with hAFS-CM/hMSC-CM using the following solution: 1 μM ZSTK474 (pan class I-PI3K inhibitor); 100 nM GDC-0941 (PI3Kα and PI3Kδ inhibitor) and 200 nM TGX-221 (PI3Kβ inhibitor).

### 2.4. Analysis of Intracellular Dox Fluorescence and Evaluation of Cell Apoptosis In Vitro

Live mNVCM cultured on coverslips were analyzed under an SP2-AOBS confocal microscope (Leica Microsystems, Wetzlar, Germany). Dox fluorescence of drug-treated cultures was analyzed in the 545–610 nm range after excitation by the 488 nm Argon laser line. Eight to ten randomly selected fields containing at least 5–8 cells were analyzed for each sample in order to quantify single-cell Dox fluorescence using specialized Leica software.

mNVCM apoptosis was measured after Dox treatment for 21 h via immunocytochemistry for the expression of cleaved caspase-3 (CC-3) enzyme. Briefly, cells were fixed with 4% PFA for 10 min, followed by incubation with 3% H_2_O_2_ for 5 min at room temperature. Cells were then incubated with a primary rabbit anti-mouse cleaved caspase-3 antibody (5A1E Cell Signaling Technology, Euroclone, Milan, Italy) overnight and then with a secondary anti-rabbit DAKO EnVision+ System HRP-labelled polymer antibody (Dako, Agilent, Santa Clara, CA, USA). Images were acquired with an Axiovert microscope equipped with Axiovision software (Carl Zeiss, Milan, Italy). CC-3-positive cells were analyzed using the plug-in cell counter from ImageJ (imagej.nih.gov/ij/) as the percentage over total cells from all regions of interest (ROI) was considered.

4T1 cell viability was evaluated by the MTT assay. Briefly, 10,000 cells/well were seeded in a 96-well plate. After 24 h, the 4T1 cells were primed with hAFS-CM and hMSC-CM and then subjected to Dox exposure. Cell viability was evaluated 21 h after Dox treatment with or without secretome priming. Cells were washed in PBS and incubated with a 150 μg/mL MTT solution (Sigma-Aldrich, Merck, Darmstadt, Germany) for 4 h. Data was acquired on a VersaMax (GE Intelligent Platforms, Charlottesville, VA, USA) plate reader.

### 2.5. Analysis of mNVCM Mitochondrial Organization and Their In Vitro Transmembrane Potential

The mitochondrial transmembrane potential of mNVCM grown coverslips was evaluated after 16 h of Dox exposure by confocal microscopy. Cells were stained with 300 nM MitoTracker™ Deep Red (Thermo Fisher Scientific, Monza, Italy) for 10 min at 37 °C and analyzed on a SP2-AOBS confocal microscope. The Helion/Neon 633 nm laser was used for excitation, and fluorescence emission was collected in the 645–750 nm range. Eight to ten randomly selected fields containing at least 5–8 cells were analyzed for each sample by dedicated Leica software for the quantitation of single-cell mitochondrial fluorescence. Cardiac fibroblasts, if present in the cell cultures, were negatively selected by anti-Thy1/CD90 (Biolegend, San Diego, CA, USA) staining and thus gated out during analysis. Nevertheless, these fibroblasts could be easily discriminated from myocytes, due to their much lower mitochondrial transmembrane potential (≈20–25% of mNVCM mitochondrial potential).

### 2.6. Biochemical Profiling on mNVCM and mNVFib

#### 2.6.1. Assessment of Oxygen Consumption Rate (OCR)

O_2_ consumption was evaluated in mNVCM and mNVFib after 6 h of Dox exposure by a thermostatically-controlled oxygraph apparatus equipped with an amperometric electrode (Unisense-Microrespiration, Unisense A/S, Aarhus, Denmark). For each experiment, 50,000 cells permeabilized with 0.03% digitonin for 10 min were employed. The electrode was equilibrated with the respiration medium containing: 137 mM NaCl, 5 mM KCl, 0.7 mM KH_2_PO_4_, 25 mM Tris–HCl pH 7.4, and 25 mg/mL ampicillin. A Hamilton syringe was used to add the respiring substrates. 10 mM pyruvate + 5 mM malate or 20 mM succinate were used to induce the pathways composed of Complexes I, III, and IV or Complexes II, III, and IV, respectively. After the addition of respiratory substrates, 0.1 mM ADP was added in each experiment. The respiratory rates are expressed as nmol O/min/10^6^ cells [26].

#### 2.6.2. Assay for Fo-F1 ATP Synthase Activity

F_o_-F_1_ ATP synthase activity was evaluated in mNVCM and mNVFib. For each experiment, 50,000 cells were incubated for 10 min at 37 °C in a medium containing: 10 mM Tris-HCl pH 7.4, 100 mM KCl, 5 mM KH_2_PO_4_, 1 mM EGTA, 2.5 mM EDTA, and 5 mM MgCl_2_, 0.6 mM ouabain, 0.3 mM P1,P5-Di(adenosine-5′) pentaphosphate, and 25 mg/mL ampicillin. Afterward, ATP synthesis was induced by the addition of 10 mM pyruvate plus 5 mM malate or 20 mM succinate, to stimulate the pathways composed of Complexes I, III, and IV or Complexes II, III, and IV, respectively. The reaction was monitored every 30 s for up to two minutes in a luminometer (GloMax^®^ 20/20n Luminometer, Promega Italia, Milan, Italy), by the luciferin/luciferase chemiluminescent method, with ATP standard solutions between 10^−8^ and 10^−5^ M (luciferin/luciferase ATP bioluminescence assay kit CLSII, Roche, Milan, Italy). Data are expressed as nmol ATP produced/min/10^6^ cells [27].

#### 2.6.3. Evaluation of Oxidative Phosphorylation Efficiency

The oxidative phosphorylation (OxPhos) efficiency was calculated as the ratio between the concentration of ATP produced and the amount of consumed oxygen in the presence of the respiring substrate and ADP (P/O ratio). When the oxygen consumption is completely devoted to energy production, the P/O ratio should be approximately 2.5 and 1.5 after pyruvate + malate or succinate addition, respectively [28].

#### 2.6.4. Glucose Consumption Assay

To evaluate the glucose consumption, the glucose content in the growth medium was evaluated by the hexokinase (HK) and glucose-6-phosphate dehydrogenase (G6PD) coupling system, following the reduction of NADP at 340 nm. The assay medium contained: 100 mM Tris-HCl pH 7.4, 2 mM ATP, 10 mM NADP, 2 mM MgCl_2_, 2 IU of hexokinase, and 2 IU of glucose-6-phosphate dehydrogenase. The data were normalized to the cell number and expressed as mM glucose consumed/10^6^ cells [29].

#### 2.6.5. Lactate Release Assay

The lactate concentration was assayed spectrophotometrically in the growth medium, following the reduction of NAD^+^ at 340 nm. The assay medium contained: 100 mM Tris-HCl pH 8, 5 mM NAD^+^, and 1 IU/mL of lactate dehydrogenase. Samples were analyzed before and after the addition of 4 μg of purified lactate dehydrogenase. The data were normalized to the cell number and expressed as mM lactate released/10^6^ cells [30].

### 2.7. Preclinical Model of Dox-Induced Cardiomyopathy

#### 2.7.1. In Vivo Model

In vivo treatments with Dox were carried out according to a previously published protocol [19]. Briefly, 2-month-old Balb/c females were treated with a cumulative dose of 12 mg/Kg Dox via 3 weekly intraperitoneal (i.p.) injections (4 mg/kg on days 0, 7, and 14). hAFS and hMSC (100 μg/mouse) were administered via i.p. 3 h before each Dox injection. Cardiac function was assessed by echocardiography, prior to and 6 weeks after the first Dox injection, in anesthetized mice (1% isoflurane) with a Vevo 2100 High-Resolution Imaging System (Visual Sonics Inc., Toronto, ON, Canada) equipped with a 30-MHz probe (MS550D, VisualSonics, Toronto, ON, Canada). Echocardiographic parameters were measured under the long-axis M-mode when the heart rate was about 450 bpm and the following parameters were evaluated, as previously described [19]: fractional shortening (FS), ejection fraction (EF), left ventricular end-diastolic diameter (LVEDd) and end-systolic diameter (LVEDs), interventricular septal thickness at end-diastole (IVSd) and at end-systole (IVSs); left ventricular posterior wall thickness at end-diastole (LVPWd) and at end-systole (LVPWs). Left ventricular (LV) weight determined by echocardiography measurements in M-mode was normalized to tibial length (TL) and used to compare relative heart mass among all groups. Body weight was monitored weekly during the entire duration of the treatment.

#### 2.7.2. Gene Expression Analysis

Total RNA from snap-frozen mouse cardiac tissue was extracted using the Qiazol Lysis Reagent (Qiagen, Milan, Italy); cDNA was obtained using the iScriptTM cDNA Synthesis Kit (Bio-Rad, Hercules, CA, USA). Real-time qRT-PCR was carried out on a 7500 Fast Real-Time PCR System (Thermo Fisher Scientific, Milan, Italy) using BrightGreen 2X qPCR MasterMix-Low ROX (Abm, Richmond, BC, Canada). Primer sequences (TibMolBiol, Genova, Italy) for murine *Anp* (Atrial natriuretic peptide), *Bnp* (Brain natriuretic peptide), *Ctgf* (Connective tissue growth factor), and *β2M* (Beta-2 Microglobulin) were available on request. Gene expression levels were normalized using *β2M* as an endogenous control by applying the 2^−ddCt^ method and considering sham healthy mice as the fold change reference.

#### 2.7.3. Evaluation of Aerobic Metabolism Alterations, Lipid Peroxidation, and Antioxidant Enzymatic Defenses

To evaluate the effect of Dox on cardiac tissue mitochondrial metabolism, the activity of Complex I (NADH-ubiquinone oxidoreductase) was assayed on 50 μg of total protein from mouse heart tissue lysate, following the ferricyanide reduction, at 420 nm. The assay medium contained 10 mM phosphate buffer pH 7.2, 30 mM NADH, 40 mM ferricyanide, and 40 μM antimycin A [30]. To analyze oxidative stress and the antioxidant enzymatic defenses, malondialdehyde (MDA) levels (as a marker of lipid peroxidation) and the activity of glucose-6-phosphate dehydrogenase (G6PD), glutathione reductase (GRx), and catalase (CAT) were evaluated. MDA concentration was assayed by the thiobarbituric acid reactive substances (TBARS) assay. Briefly, 600 μL of TBARS solution was added to 50 μg of total protein dissolved in 300 μL of Milli-Q water. The mix was incubated for 40 min at 100 °C. Afterwards, the sample was centrifuged at 14,000 rpm for 2 min and the supernatant was analyzed at 532 nm using a spectrophotometer [31]. The G6PD activity was evaluated following the NADP reduction at 340 nm. The assay mixture contained: 100 mM Tris HCl pH 7.5, 5 mM MgCl_2_, 10 mM glucose-6-phosphate, and 0.5 mM NADP [32]. GRx activity was analyzed at 405 nm using a spectrophotometer, with the Glutathione Reductase Assay Kit (Abcam, Cambridge, UK) following the manufacturer’s instructions. Catalase activity was assayed with a spectrophotometer, following the decomposition of H_2_O_2_ at 240 nm. The assay mix contained: 50 mM phosphate buffer pH 7.0, and 5 mM H_2_O_2_ [31].

### 2.8. Statistical Analyses

Results are presented as the mean ± standard error of the mean (SEM) of at least three (*n* = 3) independent replicated experiments (in vitro analyses) or independent animals (in vivo study). Comparisons were drawn by two-way repeated-measures ANOVA with the Tukey’s post-hoc tests (for the in vivo cardiac function evaluation by echocardiography), or one-way ANOVA followed by post-hoc Tukey’s multiple tests, or by an unpaired t-test (when appropriate) and analyzed by Prism Version 6.0a GraphPad Software (www.graphpad.com) with statistical significance defined as * *p* < 0.05.

## 3. Results

### 3.1. hAFS and hMSC Secretome Priming Preserves mNVCM from Unspecific Dox Uptake and from Dox-Induced Apoptosis

**Figure 1:**
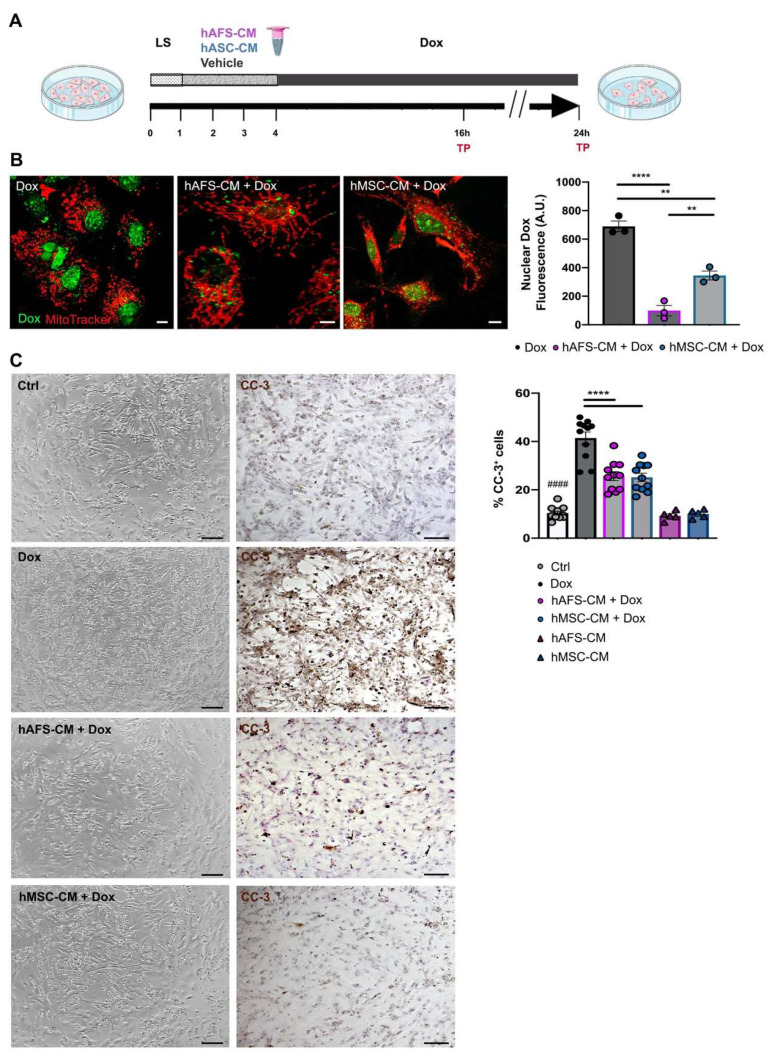
hAFS and hMSC secretome formulations exert cardioprotective effects on mNVCM exposed to Dox. (**A**) Schematic of the experimental design for in vitro experiments. (**B**) Left panel: representative confocal images of live mNVCM exposed to 1 µM Dox for 16 h with or without (*Dox*) prior priming with hAFS-CM (*hAFS-CM + Dox*) or hMSC-CM (*hMSC-CM + Dox*) for 3 h. Mitochondria are represented in red by the MitoTracker signal; Dox nuclear localization is represented in green by means of the drug auto-fluorescent signal, scale bar: 5 µm. Right panel: evaluation of Dox nuclear fluorescence by arbitrary units (*A.U.*) in Dox-exposed mNVCM (*Dox*: *689.67* ± *37.19*) versus mNVCM primed with either hAFS-CM (*hAFS-CM + Dox*: *99.33* ± *36.22*; **** *p* < 0.0001) or hMSC-CM (*hMSC-CM + Dox*: *345.33* ± *31.15*; ** *p* = 0.0011). Values are expressed as the mean ± s.e.m. of *n* = 3 independent experiments. *hAFS-CM + Dox* vs *hMSC-CM + Dox* ** *p* = 0.0060. (**C**) Left panel: representative brightfield microscopy images and immunocytochemistry analysis of the cleaved-caspase 3 enzyme (*CC-3*) on untreated mNVCM (*Ctrl*) and on mNVCM exposed for 21 h to 1 µM Dox with or without (*Dox*) previous priming with hAFS-CM (*hAFS-CM + Dox*) or hMSC-CM (*hMSC-CM + Dox*) for 3 h, scale bar: 20 μm. Left panel: percentage of mNVCM expressing cleaved-caspase-3 (*%CC-3^+^ cells*) in control condition (*n* = 11, *Ctrl: 10.35* ± *0.80%*), after either hAFS-CM (*n* = 5, *hAFS-CM: 9.24* ± *0.84%*) or hMSC-CM (*n* = 5, *hMSC-CM: 10.01 ± 0.76%*) stimulation alone or after exposure to Dox with or without (*n* = 11, *Dox: 41.43* ± *2.50%*) pre-incubation with hAFS-CM (*n* = 11, *hAFS-CM+Dox: 25.67* ± *1.83%*) or hMSC-CM (*n* = 11, *hMSC-CM + Dox: 25.16* ± *1.72%*). Values are expressed as the mean ± SEM of the independent experiments; *hAFS-CM* vs. *Ctrl* and *hMSC-CM* vs. *Ctrl*: not significant; *Ctrl* vs. *Dox;* ^####^ *p* < 0.0001; *hAFS-CM + Dox vs Dox and hMSC-CM + Dox* vs. *Dox* **** *p* < 0.0001.

A well-established in vitro model of Dox cardiotoxicity [21] was used to evaluate the effects exerted by the conditioned media released by both hAFS and hMSC undergoing hypoxic preconditioning. mNVCM, primed or not for 3 h with either hAFS-CM or hMSC-CM, were exposed to Dox (as illustrated in schematic in Figure 1A).

First, the endogenous fluorescence emitted by Dox was exploited to analyze the uptake of Dox by mNVCM. It was possible to demonstrate that mNVCM priming with either hAFS-CM or hMSC-CM induced a 6.9-fold (**** *p* < 0.0001) or 2-fold (** *p* < 0.01) reduction of nuclear Dox-associated fluorescence, when compared to the Dox-treated cells (Figure 1B), with the hAFS secretome showing more pronounced influence over the hMSC counterpart (** *p* < 0.01). Although nuclear Dox concentrations might not be strictly proportional to nuclear Dox autofluorescence, due to the fluorescence quenching of the DNA-bound fraction of Dox, our data may indicate a relevant reduction of nuclear Dox accumulation by secretome priming. While hAFS-CM confirmed to induce significant upregulation of the efflux transported *Abcb1* transcription in mNVCM (Dox vs. Dox + hAFS-CM and untreated control—Ctrl- vs. Dox + hAFS-CM, * *p* < 0.05, Figure S1B), as previously shown [21], this evidence may not be sufficient to explain the prompt extrusion of Dox from mNVCM nuclei, and therefore requires further investigation.

Next, we evaluated Dox-induced apoptosis. A 21 h incubation with Dox resulted in a significantly higher percentage of apoptotic mNVCM, as revealed by cleaved caspase-3 expression, compared to either untreated healthy cells (Ctrl, ^####^ *p* < 0.0001) or cells primed with the stem cell secretomes (**** *p* < 0.0001). The rate of apoptotic cells was significantly and similarly reduced by 37.6% and 39.3%, respectively, by pre-treatment with either hAFS-CM or hMSC-CM. hAFS and hMSC secretome formulations alone did not affect mNVCM viability (Figure 1C).

We have previously shown that the antagonism to Dox toxicity by hAFS-CM is mediated by PI3K [21], a superfamily of enzymes including 3 classes, with class I PI3K further subdivided into four members (-α, -β, -γ, and -δ isoforms) [33]. In order to evaluate whether the cardioprotective potential of the hAFS or hMSC secretomes could be mediated by a specific PI3K isoform, the combined effect of Dox and PI3K inhibitors, namely ZSTK474 (pan class I PI3K inhibitor), GDC-0941 (PI3Kα and PI3Kδ inhibitor), and TGX-221 (PI3Kβ inhibitor), were investigated. While the hAFS-secretome pro-survival effect on mNVCM exposed to Dox was maintained only in combination with the PI3Kβ inhibitor TGX-221 (* *p* < 0.05, Supplementary Figure S1A), the administration of hMSC-CM with any PI3K antagonist did not exert any cardioprotective influence (Supplementary Figure S1A). Likewise, we have reported that hAFS-CM may counteract Dox toxicity via a PI3K/Akt-dependent role for NF-κB and its target genes, *Il6* and *Cxcl1*, on mNVCM in vitro [21]. This data was here confirmed by real-time qRT-PCR when mNVCM were primed with the hAFS secretome (*Il6*: Dox vs. hAFS-CM + Dox, **** *p* < 0.0001; *Cxcl1*: Dox vs. hAFS-CM + Dox, * *p* < 0.05), but not when stimulating cardiomyocytes with hMSC-CM (Supplementary Figure S1B). Overall, these data demonstrate that the hAFS and hMSC secretomes protect mNVCM against Dox apoptosis by activating PI3K-dependent pro-survival signaling. The paracrine effects exerted by hAFS-CM and hMSC-CM were also evaluated on the 4T1 breast cancer cell line in combination with Dox exposure to analyze putative interfering effects on tumor cells during the chemotherapy regime. Priming with both the hAFS- or hMSC secretome did not induce a protective effect on 4T1 cell viability during Dox exposure. 4T1 cells exposed to Dox showed a decrease in cell viability by approximately 23.49% versus the untreated cells (Ctrl vs Dox, ^####^ *p* < 0.0001, Figure S1D). Both hAFS- and hMSC-CM did not interfere with the effect of Dox (*p* > 0.05). Likewise, 4T1 cells primed by both secretomes did not result in an increase in cell proliferation, when compared to the untreated condition (*p* > 0.05), suggesting that while hAFS-CM and hMSC-CM are cardioprotective, they have no stimulatory influence on Dox-sensitive breast cancer cells.

### 3.2. The Paracrine Potential of hAFS- and hMSC-CM Counteracts Dox-Induced Mitochondrial Dysfunction on mNVCM

**Figure 2:**
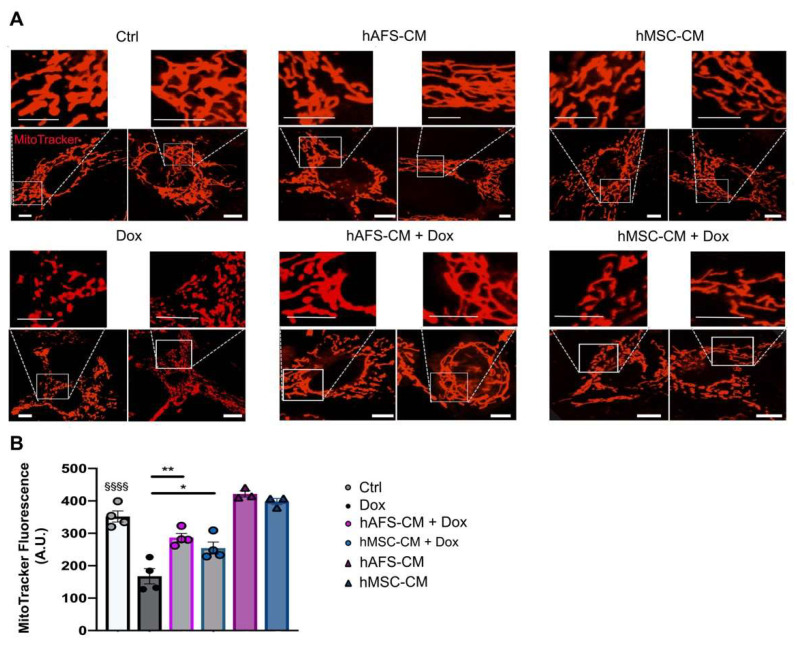
hAFS and hMSC secretomes preserve mitochondrial transmembrane potential in mNVCM exposed to Dox. (**A**) Representative confocal images of live mNVCM in control condition (*Ctrl*), undergoing stimulation with either hAFS-CM (*hAFS-CM*) or hMSC-CM (*hMSC-CM*) for 3 h or exposed to 1 µM Dox for 16 h with or without (*Dox*) prior priming with hAFS-CM (*hAFS-CM + Dox*) or hMSC-CM (*hMSC-CM + Dox*) for 3 h. The cell mitochondrial network is represented in red by the MitoTracker signal, scale bar: 5 µm, (**B**) Mitochondrial transmembrane potential evaluated by MitoTracker fluorescence analysis and expressed by arbitrary units (*A.U.*) mNVCM treated as follows: control condition (*n* = 4, *Ctrl: 351.85* ± *17.13*); after exposure to 1 μM Dox for 16 h (*n* = 4, *Dox: 167.52* ± *23.49*); pretreated with hAFS-CM or hMSC-CM for 3 h before 1 μM Dox stimulation for 16 h (*n* = 4, *hAFS-CM + Dox: 286.77* ± *12.94,* and *hMSC-CM + Dox: 254.57* ± *18.56*, respectively), or only pretreated with hAFS-CM or hMSC-CM (*n* = 3, *hAFS-CM: 421.63* ± *9.55* and *hMSC-CM: 397.93* ± *9.57*, respectively). Values are expressed as the mean ± SEM of the independent experiments. *hAFS-CM* vs. *Ctrl* and *hMSC-CM* vs. *Ctrl*: not significant; *Ctrl* vs. *Dox;* ^§§§§^ *p* < 0.0001; *hAFS-CM + Dox* vs. *Dox* ** *p* = 0.0012*; hMSC-CM +Dox* vs. *Dox* * *p* = 0.0185.

Confocal microscopy studies of Dox-treated mNVCM stained with MitoTracker showed increased mitochondrial hyper-fission with breakage of the mitochondrial network and decreased mitochondrial transmembrane potential. Interestingly, the pattern of the mitochondrial network was preserved upon pre-treatment with both CM (Figure 2A). Dox treatment also decreased the mitochondrial transmembrane potential, as revealed by the average intracellular MitoTracker fluorescence. However, the Dox-induced drop of mitochondrial transmembrane potential was partially hindered by the presence of the hAFS or hMSC secretome (Figure 2B).

**Figure 3:**
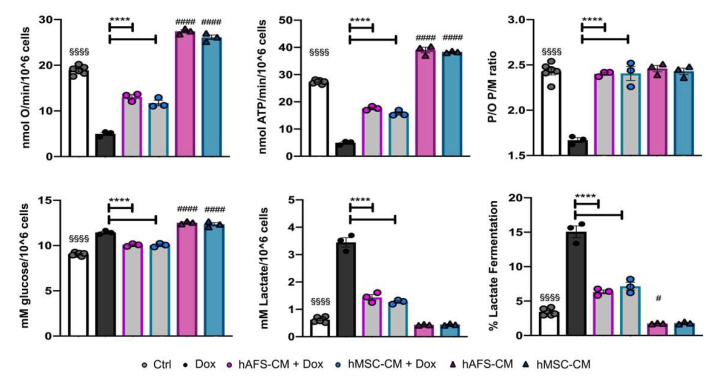
Dox-elicited mitochondrial dysfunction is rescued in mNVCM pretreated with either hAFS-CM or hMSC-CM. From the top left corner: Oxygen consumption rate stimulated with pyruvate plus malate; ATP production by F_o_-F_1_ ATP synthase stimulated with pyruvate plus malate; mNVCM P/O ratio after pyruvate plus malate stimulation, as a marker of OxPhos efficiency; Glucose consumption; lactate release; lactate fermentation percentage. All the biochemical measurements were performed in mNVCM treated as follows: control condition (*Ctrl*), after exposure to 1 µM Dox (*Dox*), pretreated with hAFS-CM or hMSC-CM before the Dox stimulation (*hAFS-CM+Dox* and *hMSC-CM + Dox*, respectively), or only pretreated with hAFS-CM or hMSC-CM (HAFS-CM and hMSC-CM, respectively). In each panel, values are expressed as the mean ± SEM of the independent experiments; *hAFS-CM* vs. *Ctrl* and *hMSC-CM* vs. *Ctrl* ^#^ *p* = 0.0495 or ^####^ *p* < 0.0001; *Ctrl* vs. *Dox* ^§§§§^ *p* < 0.0001; *hAFS-CM + Dox* vs. *Dox and hMSC-CM + Dox* vs. *Dox* **** *p* < 0.0001.

The altered mitochondrial transmembrane potential due to Dox treatment was confirmed by evaluating the oxidative phosphorylation (OxPhos) activity. Dox-treated mNVCM showed a 3-fold decrement of the oxygen consumption rate (OCR) and a 4-fold reduction of ATP synthesis in comparison to the untreated sample, after OxPhos activity was induced by pyruvate plus malate (Figure 3 and Table S1). Moreover, Dox-treated mNVCM were characterized by an inefficient ATP production, as demonstrated by a 1.5-fold reduction of the P/O ratio value, indicating an uncoupled status between respiration and energy production (Figure 3, Supplementary Figure S1C, Table S1). Conversely, the pre-treatment with hAFS-CM or hMSC-CM partially reverted the Dox-induced dysfunction of OCR and ATP synthesis (**** *p* < 0.0001, for both hAFS-CM and hMSC-CM), completely restoring the OxPhos efficiency (**** *p* < 0.001, for both hAFS-CM and hMSC-CM, Figure 3 and Table S1). A similar trend was observed when OxPhos activity was induced by succinate (Supplementary Figure S1C).

As a consequence of the altered OxPhos, Dox-treated mNVCM showed a slight increment in glucose consumption and a 5.5-fold enhancement of lactate release, suggesting a metabolic switch to lactate fermentation. Also, in this case, the pre-treatment with both secretomes drastically reduced the anaerobic glycolysis rate (**** *p* < 0.0001 for both hAFS-CM and hMSC-CM). Interestingly, the secretome effects were also observed on the Dox-untreated mNVCM that were not treated with Dox, as shown by a 1.3-fold increase in the OxPhos activity (^####^ *p* < 0.0001, for both secretomes) and a 2-fold decrease in the anaerobic glycolysis rate (^#^ *p* < 0.05, for hAFS-CM, Figure 3 and Table S1).

### 3.3. Both hAFS- and hMSC-Secretome Preserve mNVFib from Dox-Induced Apotosis and Mitochondrial Impairment

**Figure 4:**
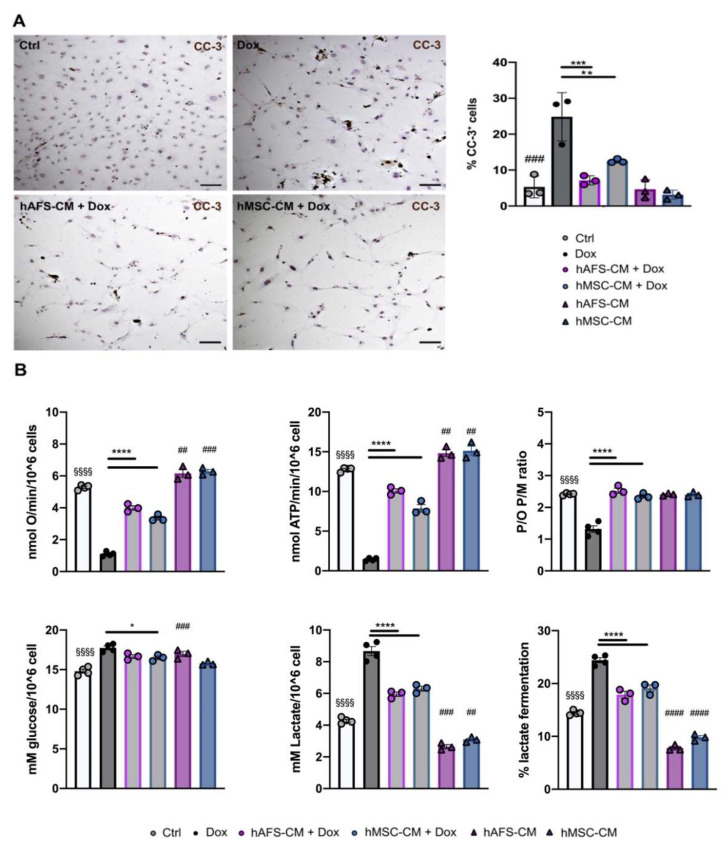
hAFS and hMSC secretomes induce pro-survival effects on mNVFib exposed to 1 μM Dox and sustain their mitochondrial metabolism. (**A**) Left panel: representative images of immunocytochemistry analysis for the expression of cleaved-caspase 3 enzyme (CC-3) on untreated mNVFib (*Ctrl*) and on mNVFib exposed for 21 h to 1 μM Dox with or without (*Dox*) previous priming with hAFS-CM (*hAFS-CM + Dox*) or hMSC-CM (*hMSC-CM + Dox*) for 3 h, scale bar: 20 µm. Right panel: percentage of mNVFib expressing cleaved-caspase-3 (%CC-3^+^ cells) in control condition (*Ctrl*: 5.31 ± 1.75%), after either hAFS-CM (*hAFS-CM*: 4.65 ± 1.54%) or hMSC-CM (*hMSC-CM*: 3.14 ± 0.74%) stimulation alone or after exposure to 1 μM Dox with or without (*Dox*: 24.83 ± 3.88%) pre-incubation with hAFS-CM (*hAFS-CM+Dox*: 7.15 ± 0.72%) or hMSC-CM (*hMSC-CM + Dox*: 12.52 ± 0.33%). Values are expressed as the mean ± SEM of *n* = 3 independent experiments; *hAFS-CM* vs. *Ctrl* and *hMSC-CM*. *Ctrl:* not significant; *Ctrl* vs. *Dox*: ^###^ *p* = 0.0001; *hAFS-CM* + *Dox* vs. *Dox*: *** *p* = 0.0003 and *hMSC-CM + Dox* vs. *Dox*: ** *p* = 0.0064. (**B**) From the top left corner: Oxygen consumption rate stimulated with pyruvate plus malate; ATP production by F_o_-F_1_ ATP synthase stimulated with pyruvate plus malate; mNVCM P/O ratio (marker of OxPhos efficiency) after pyruvate plus malate stimulation; glucose consumption; lactate release; lactate fermentation percentage. All the biochemical measurements were performed in mNVFib treated as follows: control condition (*Ctrl*), after exposure to 1 μM Dox (*Dox*), pretreated with hAFS-CM or hMSC-CM before the Dox stimulation (*hAFS-CM + Dox* and *hMSC-CM + Dox*, respectively), or only pretreated with hAFS-CM or hMSC-CM (*hAFS-CM* and *hMSC-CM*, respectively). In each panel, values are expressed as the mean ± SEM of the independent experiments*; hAFS-CM* vs. *Ctrl* ^##^ *p* < 0.01 (*p* = 0.0035 for oxygen consumption rate and *p* = 0.0015 for ATP production), ^###^ *p* < 0.001 (*p* = 0.0002 glucose consumption and for lactate release) and ^####^ *p* < 0.0001; *hMSC-CM* vs. *Ctrl* ^##^ *p* < 0.01 (*p* = 0.0018 for ATP production and *p* = 0.0032 for lactate release) ^###^ *p* < 0.001 (*p* = 0.0010 for oxygen consumption rate) and ^####^ *p* < 0.0001; *Ctrl* vs. *Dox* ^§§§§^ *p* < 0.0001; *hAFS-CM + Dox* vs. *Dox* and *hMSC-CM + Dox* vs. *Dox* **** *p* < 0.0001 and * *p* = 0.0247.

Since cardiac fibroblasts are key players during the myocardial stress response [33], we also evaluated the potential of cell secretome treatments in the detrimental effects of Dox on primary cultures of mNVFib. Following the same experimental approach already described for mNVCM in Figure 1, Dox treatment induced mNVFib apoptosis, which, although to a lesser extent when compared to mNVCM, was significantly increased by 4.6-fold compared to the control untreated cells (^###^ *p* < 0.001, Figure 4A). Pre-treatment with either hAFS-CM or hMSC-CM significantly preserved fibroblasts from Dox-induced apoptosis, reducing the percentage of apoptotic cells by 71.2% (*** *p* < 0.001) and by 49.6% (** *p* < 0.01), respectively, compared to the Dox-treated group. hAFS and hMSC secretome formulations alone did not affect mNVFib viability (Figure 4A).

The Dox-induced dysfunction of aerobic metabolism was also evaluated in mNVFib, that received or did not receive the pre-treatment with the two cell secretomes. The treatment with Dox determined a similar trend to that reported for mNVCM. In particular, when samples were stimulated with pyruvate plus malate, Dox induced a 5-fold decrement of OCR and ATP synthesis, thus determining an uncoupled status (Figure 4B and Table S1). By contrast, the pre-treatment with hAFS-CM or hMSC-CM showed a 2-fold recovery of the OxPhos activity (**** *p* < 0.0001, for both CM) and a completely restored the OxPhos efficiency (**** *p* < 0.0001, for both CM) (Figure 4B and Table S1). Similar results were observed when succinate was used to stimulate OxPhos activity (Figure S1C). Moreover, the stem cell secretomes reduced by 2-fold the Dox-induced upregulation of anaerobic glycolysis (**** *p* < 0.0001, Figure 4B and Table S1).

### 3.4. hAFS- and hMSC-CM Protect Against DOX Cardiotoxicity In Vivo

To evaluate the in vivo paracrine effects of the secretomes released by both hAFS and hMSC in antagonizing Dox-induced cardiotoxicity, Balb/c female mice were subjected to a low dose Dox administration once a week for 3 weeks, with or without concomitant i.p. delivery of hAFS-CM, hMSC-CM or vehicle saline solution a few hours before each Dox injection (as reported in the schematic in Figure 4A). As reported in a recent statement by the American Heart Association [34], the severity of the cardiovascular phenotypes induced by Dox can differ significantly according to the genetic background of the animal strain used. The choice of an apparently low cumulative dose of Dox in our study was driven by the higher vulnerability of Balb/c mice compared to other mouse strains, such as C57BL/6, to different types of cardiac stress [35,36,37]. Indeed, in Balb/c mice, the cumulative dose of 12 mg/Kg was previously shown to recapitulate the late-onset myocardial dysfunction observed clinically in patients, without triggering the cardiac-unrelated mortality that is typical of Dox regimens featuring higher cumulative doses. Moreover, the use of female mice in the study was intended to model a clinically relevant problem, which is anthracycline cardiotoxicity in the growing population of breast cancer survivors, so to provide clinical relevance of our findings. Dox induced a significant decrease in mice body weight 42 days after the initial Dox exposure (Sham vs. Dox, ^####^ *p* < 0.0001) which was significantly rescued by i.p. administration of both hAFS- and hMSC-CM with comparable outcomes (Dox vs. Dox + hAFS-CM, *** *p* < 0.001; Dox vs. Dox + hMSC-CM, ** *p* < 0.001, Figure 4B). Likewise, hAFS-CM administration counteracted long-term Dox-induced atrophy (* *p* < 0.05) by maintaining left ventricle mass/tibial length ratio comparable to the sham control group (Figure S1E).

When the cardiac function was evaluated by means of echocardiography after 6 weeks, Dox treatment was associated with a trend of decreased left ventricular posterior wall thickness at end-diastole with partial, yet significant, increase in left ventricular end-systolic diameter (Dox at day 0 vs. Dox at day 42, ^##^ *p* < 0.01, Table S2). Both fractional shortening and ejection fraction resulted affected in the Dox group in the long term (Dox at day 0 vs. Dox at day 42, ^####^ *p* < 0.0001 and ^###^ *p* < 0.001, respectively, Table S2). Co-administration of either hAFS-CM or hMSC-CM inhibited the detrimental effects of Dox resulting in significant restoration of the fractional shortening several weeks after the last administration (Dox vs. Dox+hAFS-CM and Dox vs. Dox+hMSC-CM at day 42, ** *p* < 0.01, Figure 4C and Table S2). Improvement in ejection fraction at day 42 was also detected, following i.p. delivery of the stem cell secretomes, although this was statistically significant only for hMSC-CM treatment (* *p* < 0.05, Table S2).

**Figure 5:**
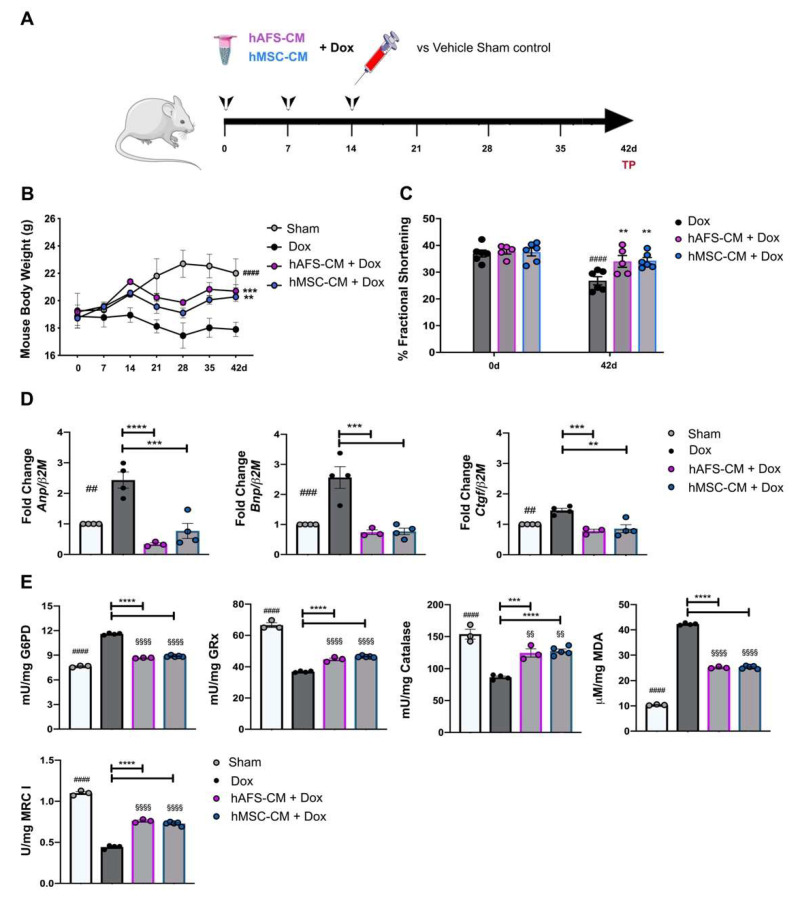
hAFS and hMSC secretomes exert long-term cardioprotective effects in a preclinical mouse model of Dox-induced cardiomyopathy. (**A**) Schematic of the experimental design for in vivo experiments; a cumulative 12 mg/Kg Dox dose was administered by intra-peritoneal delivery three times, once a week, over 14 days (4 mg/kg on days 0-7-14). The hAFS-CM and hMSC-CM (100 μg/mouse) were administered 3 h before each 4 mg/kg Dox injection. (**B**) Evaluation of mice body weight over time in sham healthy control animals (*n* = 3, *Sham*: day 0: 19.27 ± 1.27 g; day 42: 22.03 ± 1.03 g) and in mice receiving Dox with or without (*n* = 4, *Dox*: day 0: 18.85 ± 0.83 g; day 42: 17.90 ± 0.52 g) pre-incubation with hAFS-CM (*n* = 6, *hAFS-CM + Dox*: day 0: 19.16 ± 0.15 g; day 42: 20.70 ± 0.47 g) or hMSC-CM (*n* = 5, *hMSC-CM + Dox*: day 0: 18.72 ± 0.53 g; day 42: 20.28 ± 0.33 g). Values are expressed as the mean ± SEM of the different animals. *Sham* vs. *Dox* at day 42: ^####^ *p* < 0.0001; *hAFS-CM + Dox* vs. *Dox* at day 42: *** *p* = 0.0008 *and hMSC-CM +Dox* vs. *Dox* at day 42: ** *p* = 0.0083. (**C**) Ultrasound analysis of cardiac function via evaluation of fractional shortening percentage over time in mice receiving a cumulative dose of 12 mg/Kg Dox with or without (*n* = 6, *Dox*: day 0: 37.0 ± 1.30% vs. day 42: 26.80 ± 1.30%) pre-incubation with hAFS-CM (*n* = 5, *hAFS-CM + Dox*: day 0: 37.80 ± 1.02%; day 42: 34.01 ± 2.20%) or hMSC-CM (*n* = 6, *hMSC-CM + Dox*: day 0: 37.50 ± 1.44%; day 42: 34.30 ± 1.20%). Values are expressed as the mean ± SEM of the independent animals. Dox day 0 vs. day 42: ^####^ *p* < 0.0001; hAFS-CM + Dox vs. Dox at day 42: ** *p* = 0.0055 and hMSC-CM +Dox vs. Dox at day 42: ** *p* = 0.0026. (**D**) Real time qRT-PCR analysis on murine myocardial tissue 42 days after starting Dox administration with or without (*Dox*, *n* = 4) pre-treatment with hAFS-CM (*hAFS-CM + Dox*, *n* = 3) or hMSC-CM (*hMSC-CM+ Dox*, *n* = 5) versus sham healthy control mice (*Sham*, considered as calibrator, *n* = 4) for the fold-change expression of *Anp* (Atrial natriuretic peptide; *Dox*: 2.43 ± 0.22; *hAFS-CM + Dox*: 0.34 ± 0.03; *hMSC-CM+Dox*: 0.77 ± 0.21), *Bnp* (Brain natriuretic peptide; *Dox:* 2.56 ± 0.31; *hAFS-CM + Dox*: 0.74 ± 0.06; *hMSC-CM + Dox*: 0.77 ± 0.09), *Ctgf* (Connective tissue growth factor; *Dox*: 2.56 ± 0.31; *hAFS-CM + Dox*: 0.74 ± 0.06; *hMSC-CM + Dox*: 0.77 ± 0.09) over the *β2M* (Beta-2 Microglobulin) housekeeping gene. In each panel, values are expressed as the mean ± SEM of the independent experiments. *Sham* vs. *Dox*: ^##^ *p* = 0.0011 (*Anp*), ^###^ *p* = 0.0008 (*Bnp*) and ^##^ *p* = 0.0084 (*Ctgf*); *hAFS-CM + Dox* vs. *Dox*: **** *p* < 0.0001 (*Anp*), *** *p* = 0.0004 (*Bnp*), *** *p* = 0.0008 (*Ctgf)*; *hMSC-CM + Dox vs Dox*: *** *p* = 0.0003 (*Anp*), *** *p* = 0.0002 (*Bnp*) and ** *p* = 0.0012 (*Ctgf*). (**E**) From the top left corner: glucose-6-phosphate dehydrogenase (G6PD) activity; glutathione reductase (GRx) activity; catalase (CAT) activity; malondialdheyde (MDA) intracellular concentration; Complex I (MRC I) activity. All the biochemical measurements have been performed at 42 days from initial treatment in the hearts of: sham healthy control mice (*Sham*, *n* = 3), mice receiving a cumulative dose of 12 mg/Kg Dox (*Dox*, *n* = 4) with or without pre-administration of hAFS-CM (*hAFS-CM + Dox*, *n* = 3) or hMSC-CM (*hMSC-CM + Dox*, *n* = 5). In each panel, values are expressed as the mean ± SEM of the independent experiments and reported in Table S2; *Sham* vs. *Dox*: ^####^ *p* < 0.0001; *Sham* vs. *hAFS-CM*: ^§§§§^ *p* < 0.0001 and ^§§^ *p* = 0.0067 (CAT); *Sham* vs. *hMSC-CM*: ^§§§§^ *p* < 0.0001 and ^§§^ *p* = 0.0065 (CAT); *hAFS-CM + Dox* vs. *Dox*: ***** *p* < 0.0001 and *** *p* = 0.0006 (CAT); *hMSC-CM + Dox* vs. *Dox*: ***** *p* < 0.0001.

Dox administration also resulted in the long term upregulation of cardiac injury markers, with a 2.4- and 2.6-fold increase of myocardial *Anp* (^##^ *p* < 0.01) and *Bnp* (^###^ *p* < 0.001) mRNA levels, respectively, compared to the sham healthy control mice, consistently to what previously reported with the same preclinical model of chronic cardiomyopathy [38]. Conversely, in vivo treatment with both hAFS and hMSC secretomes resulted in a significant decrease *Anp* expression by 7- (**** *p* < 0.0001) and 3-fold (*** *p* < 0.001) and by 3-fold for *Bnp* (*** *p* < 0.001), respectively (Figure 5D). Profibrotic *Ctgf* was moderately increased following Dox treatment (^##^ *p* < 0.01); yet, such an effect was significantly reduced and almost restored to baseline expression by both hAFS-CM (*** *p* < 0.001) and hMSC-CM (** *p* < 0.01).

Next, we evaluated the effects of hAFS-CM or hMSC-CM administration on mitochondrial metabolism, antioxidant defenses, and lipid peroxidation accumulation in Dox-treated hearts. Data showed that Dox treatment determined a 2.5-fold reduction of respiratory Complex I (^####^ *p* < 0.0001), confirming the negative effect of Dox on the OxPhos activity. Moreover, Dox treatment determined a 1.5-fold increment of G6PD activity (^####^ *p* < 0.0001) but a 2-fold decrement of GRx and CAT activity with respect to the sample control (^####^ *p* < 0.0001, Figure 5E and Table S3), suggesting that the antioxidant defenses of cardiac tissue were not able to contrast the oxidative stress induced by anthracycline treatment. This hypothesis was confirmed by the accumulation of malondialdehyde, a marker of lipid peroxidation, which increased 4-fold compared to the untreated control (^####^ *p* < 0.0001, Figure 5E and Table S3). However, both hAFS-CM and hMSC-CM administration reverted these negative effects by restoring, at least in part, the activity of antioxidant enzymes (for both secretomes: **** *p* < 0.0001 for G6PD; *** *p* < 0.001 for GRx; *** *p* < 0.001 and **** *p* < 0.0001 for CAT) and reducing the malondialdehyde accumulation by 1.7-fold (**** *p* < 0.0001 for both secretomes).

## 4. Discussions

We previously reported that the hAFS secretome exerts significant cardioprotective and anti-senescent effects on Dox-exposed cardiomyocytes by triggering the PI3K/Akt signaling axis with subsequent modulation of *Il6* and *Cxcl1*. Here we compared the hAFS paracrine potential to that exerted by adult somatic hMSC. While the hAFS secretome was confirmed to antagonize Dox-induced cardiomyocyte apoptosis possibly via activation of the PI3Kα pathway, it was not possible to identify a corresponding specific PI3K candidate for the hMSC-CM, since all the PI3K antagonists investigated (PI3Kα/δ/β inhibitors) exerted comparable effects. The hMSC-conditioned medium did not induce upregulation in treated mNVCM of the same molecular candidates (*Abcb1b*, *Il6*, and *Cxcl1*) as did hAFS-CM, thus suggesting that fetal and adult progenitor secretomes may act on different target signaling pathways and consequently exert different cardioprotective effects.

Priming cardiomyocytes with either hAFS-CM or hMSC-CM resulted in a significant decrease in short-term nuclear localization of Dox in the short time (i.e., 16 h from initial Dox exposure). This may relate to reduced pro-apoptotic and poisoning effects on topoisomerase II beta, a Dox target within cardiomyocytes associated with anthracycline-derived DNA damage activation and mitochondrial dysfunction (as comprehensively addressed in [39]). Although we confirmed the ability of the hAFS secretome to upregulate *Abcb1b* transcription in Dox-treated cardiomyocytes, as initially shown in a previous study [21], this may not fully justify such prompt cardioprotective responses. Indeed, cardiomyocytes have been reported to upregulate P-glycoprotein transporter protein expression in vitro, following Dox stimulation for at least 48 h [38]; therefore, a further detailed investigation is required to better address this aspect.

Cardiac toxicity of anthracyclines was originally ascribed to their ability to promote the production of cytotoxic reactive oxygen species (ROS) and, in turn, cardiomyocyte death (the so-called “ROS-driven hypothesis”). Nonetheless, the modest effects of antioxidants in small clinical studies have suggested that additional mechanisms could be involved. Currently, Dox-induced cardiotoxicity is considered the result of multiple and partly intertwined cellular alterations that, in addition to ROS production and topoisomerase II poisoning, could include mitochondrial iron accumulation, mitochondrial biogenesis disruption, calcium disturbances, as well as alterations of cell survival pathways [40]. Therefore, here we focused on the cardiomyocyte mitochondria as a common therapeutic target of the paracrine potential of both stem cell secretome formulations.

Mitochondrial homeostasis represents a critical aspect for ensuring proper cardiac function and performance. Deregulated cardiomyocyte mitochondrial activity has been linked to myocardial dysfunction, cardiac disease, and aging [41,42,43]. Likewise, the mechanisms underlying the cardiotoxic side effects of anthracycline-based anticancer treatments have been reported to converge on cardiomyocyte mitochondrial derangement. Indeed, Dox has been reported to strongly affect both the structure and the function of cardiac mitochondria, resulting in metabolism disruption, and in the activation of different pathways leading to cardiomyocyte death and Dox-induced cardiomyopathy [44]. Since mitochondrial impairment is a crucial trigger of cardiovascular pathogenesis, intensive research efforts are addressing mitochondria homeostasis as an explicit therapeutic approach and a working strategy for the treatment of anthracycline-based cardiotoxicity.

In response to Dox injury, mitochondrial network dynamics and mitochondrial metabolism of mNVCM were significantly affected, whereby we found an induced metabolic switch towards anaerobic glycolysis. Conversely, priming with both hAFS and hMSC secretomes preserved cardiomyocyte mitochondria reticulum organization, restored the OxPhos function and efficiency, and reduced the anaerobic glycolytic rate. This amelioration could depend on both a direct effect on the Dox-induced impairment of the electron transport chain but also on the improvement of the mitochondrial network. In fact, it is known that Dox determines a reduction of the mRNA expression of Reiske iron-sulfur protein (RISP), a ubiquitous electron transport chain component [45]. However, aerobic metabolism is more efficient when mitochondria are organized in a reticulum [46,47], which allows for better communication among mitochondria and other cellular membrane-bound, necessary for the maintenance of cellular homeostasis [48,49,50]. In this regard, efficient removal of the Dox-injured mitochondria through autophagy is pivotal for preserving mitochondrial homeostasis and metabolism in the heart and, in turn, for preventing Dox cardiotoxicity [19]. Therefore, activation of mitochondrial autophagy could represent an alternative mechanism by which hAFS and hMSC secretomes preserve mitochondrial metabolism and cardiac function in response to Dox.

Interestingly, similar protective actions of both secretomes were observed in cardiac fibroblasts exposed to Dox. Cardiac fibroblasts represent a crucial cell type in orchestrating cardiac maturation and cardiac repair mechanisms and mNVCM primary culture also include a fibroblast component. This prompted us to investigate the in vitro stem cell cardioprotective effects on a more homogeneous neonatal mouse cardiac fibroblast preparation (mNVFib). Although mNVFib proved to be more resistant to Dox-induced apoptosis, they showed a similar response to the cardioprotective and pro-survival priming from either hAFS- or hMSC-CM. From the metabolic perspective, OxPhos activity of mNVFib appeared impaired by Dox treatment similar to that observed in mNVCM; priming cells with the hAFS and hMSC secretomes resulted in a positive effect on aerobic metabolism on the Dox-untreated mNVFib, although the effects were less evident in comparison to those observed in cardiomyocytes. However, our data show that mNVFib displayed lower aerobic metabolism when compared to mNVCM, making it possible to speculate that the mNVFib contribution to the oxidative stress associated with the dysfunctional OxPhos activity is marginal with respect to that observed in Dox-treated mNVCM.

Notably, when both the hAFS- and hMSC secretomes were used to prime a Dox-sensitive cancer cell line, they failed to rescue their viability. Such evidence may suggest that hAFS-CM and hMSC-CM may mediate pro-survival effects when acting on cells with very limited proliferative potential—such as cardiomyocytes—by antagonizing Dox pro-apoptotic influence. However, when Dox affected cell viability by actively interfering with DNA replication and cell cycle progression, both secretomes were not effective. Therefore, we may exclude a jeopardizing effect of both fetal and adult stromal secretomes in affecting anti-cancer Dox chemotherapy while protecting the myocardial tissue. These findings are in agreement with previously reported evidence showing that hAFS-CM promoted *Abcb1b* transcription and Dox efflux from cardiomyocytes, but it was not able to do the same in the MDA-MB-231 human breast cancer (adenocarcinoma) cell line treated with the same protocol [21].

The cardioprotective action of both hAFS- or hMSC-CM could be recapitulated in vivo in a clinically relevant murine model of Dox cardiomyopathy, reminiscent of that observed in cancer survivors who usually manifest cardiotoxicity long after therapy completion [13]. Our results showed that early i.p. administration of both hAFS- and hMSC-CM during Dox treatment exerted beneficial long-term effects resulting in the preservation of cardiac function and body mass, while they counteracted signs of cardiac fibrosis and myocardial dysfunction up to several weeks after initial priming. This further confirms previous data on the cardio-active influence of the hAFS secretome in reactivating endogenous mechanisms of repair and regeneration by providing pro-survival, anti-fibrotic, and proliferative effects on the injured myocardium in a preclinical mouse model of myocardial infarction, up to 4 weeks after local delivery [23]. This prolonged beneficial effect in vivo provides a proof of principle for a stem cell secretome-based paracrine therapy to inhibit, or at least delay, Dox-elicited cardiomyopathy. In such a perspective, the stem cell paracrine cardioprotective approach may be envisioned as a therapeutic strategy to be combined with standard oncological anthracycline regimens to most cancer patients, including pediatric ones for whom enduring cardiotoxicity side effects may represent a harmful threatening complication during adulthood, increasing the risk of developing heart failure.

The paracrine contribution of stem/progenitor cells in antagonizing Dox detrimental effects on cardiac cells has been recently evaluated after transplantation of embryonic stem cells (ES) or induced pluripotent stem cells (iPS) and their secretome formulations in preclinical cardiotoxicity models [51,52,53,54,55]. While such reports provide encouraging results in showing that both the cells and their secretome significantly decreased the adverse pathological phenotype of Dox-induced cardiomyopathy, clinical translation of ES and/or iPS may be hampered by ethical concerns and by the time-consuming and cost-effective limitations in their use. Here we evaluated two different stem cell sources; more specifically, their ability to preserve cardiomyocyte mitochondrial structure and metabolism has been considered in order to define the most suitable progenitor for further translation into the clinical scenario as a “paracrine hub” for the scale-up production of cell-free, off-the-shelf secretome pharmaceutical formulations. Indeed, both adult adipose tissue and amniotic fluid represent promising discarded backup sources of MSCs thanks to their abundance, accessibility, and less invasive collection when compared to other MSC sources such as bone marrow. In several cell-based pre-clinical approaches, MSCs from different sources have been regarded as suitable tools for preventing Dox cardiomyopathy. However, many issues remain to be elucidated in understanding the paracrine mechanisms of action of MSC and the selection of the optimal source. Indeed, just as in certain pathologies there may be the advantage of using one cell population over another, accordingly, it may be important to know in depth the functional differences between the corresponding secretomes to be used in cell-free therapeutic approaches. In this context, whilst both hAFS and hMSC exerted similar beneficial cardioprotective paracrine effects on compromised cardiomyocytes, adult progenitors may represent a cell option that is quite likely to be affected by low yield and limited self-renewal, as they are often influenced by donor age [56,57,58]. On the contrary, hAFS may offer a more feasible alternative as being an easily accessible source of fetal progenitors free from any ethical concern and available in large amounts as leftover material during prenatal screening or at the term of gestation as clinical waste during scheduled C-section delivery. Indeed, the use of fetal stem cells or their secretome formulations for downstream therapeutic applications could reduce some of the disadvantages of limited stem cell potential associated with adult stem cells. Notably, despite hAFS and hMSC being comparable in preserving mitochondrial function in our Dox-elicited cardiotoxicity model, we further confirmed that only the hAFS secretome induced cardiomyocytes to upregulate the expression of pro-survival genes *Abcb1b, Il6,* and *Cxcl1* [21] over the hMSC one; likewise, priming cardiomyocytes with the hAFS-CM resulted in a more pronounced effect in decreasing unspecific Dox intracellular uptake.

## 5. Conclusions

Overall, here we demonstrated that the hMSC secretome and, most of all the hAFS secretome, may represent feasible and exploitable options for future paracrine cardioprotective priming therapy against Dox-related cardiotoxicity during cancer treatment.

While the present study offers encouraging results in suggesting the application of the fetal and adult stem cell secretomes as a paracrine, cell-free cardioprotective strategy in combination with standard chemotherapy, some limitations must be acknowledged and need to be addressed with further research.

While the hAFS- and hMSC secretomes were not able to positively affect a Dox-sensitive cancer cell line in vitro, we believe that additional in-depth analyses are needed to confirm such aspects in human cancer lines, as well as in preclinical xenograft tumor models. In our in vitro study, different wild-type mouse strains were used as a source of cardiac cells. Although it is very unlikely that this may have influenced cardiomyocyte and/or cardiac fibroblast response to secretome priming and Dox exposure, further studies are required to confirm results from primary cell cultures obtained from the same strains considered for the in vivo preclinical model of anthracycline-driven cardiotoxicity.

Moreover, the influences specifically exerted by either hAFS- or hMSC-derived extracellular vesicles on the Dox-injured cardiomyocytes and cardiac tissue should be investigated via ad hoc potency assays, in order to dissect which stem cell secretome formulation may represent the most suitable one for future clinical application. Likewise, combinatorial formulations of both secretomes should be investigated to assess potential synergistic effects.

Resident cardiac macrophages have been recently highlighted as critical regulators of cardiomyocyte homeostasis via their significant contribution to mitophagy and mitochondria quality surveillance [59]; thus, a detailed analysis of the paracrine modulation of cardiac macrophages exposed to Dox and under the hAFS- and hMSC-CM instruction could offer useful insights to define additional target candidates for a cardioprotective strategy.

Finally, the comprehensive characterization of the intimate connection between the cardiomyocytes and cardiac fibroblasts primed with the stem cell secretome prior to Dox exposure will provide relevant information in terms of their synergistic cooperation over independent stimulation. Since alteration in the crosstalk between cardiomyocytes and the surrounding stromal cells may contribute to myocardial dysfunction, dissecting their paracrine relationship may help in tailoring combined therapeutic approaches to promptly counteract Dox detrimental effects and avoid the further development of cardiotoxic cardiomyopathy and heart failure.

## Data Availability

The data presented in this study are available in main text and supplementary materials.

## Supplementary Materials

The following are available at https://www.mdpi.com/article/10.3390/cancers13153729/s1. Table S1: detailed values of biochemical analyses on mNVCM and mNVFib exposed to Dox, with or without hAFS-CM and hMSC-CM priming; values are expressed as the mean ± SEM of at least *n* = 3 independent experiments and refers to graphs shown in Figure 2 and Figure 3, Table S2: Left ventricle functional parameters in mice exposed to Dox, with or without hAFS-CM and hMSC-CM priming. Values are expressed as the mean ± SEM from echocardiography evaluation and are shown in Figure 5, Table S3: detailed values referring to biochemical analyses on the cardiac tissue of mice treated with Dox after hAFS-CM and hMSC-CM priming. Values are expressed as the mean ± SEM of at least *n* = 3 independent experiments and refer to data in Figure 5, Figure S1: supplementary in vitro analysis on mNVCM apoptosis and gene expression and biochemical profiling of mNVCM and mNVFib.
