# Supplementary material for: The Human Fetal and Adult Stem Cell Secretome Can Exert Cardioprotective Paracrine Effects against Cardiotoxicity and Oxidative Stress from Cancer Treatment
Source: Cancers (Basel). 2021 Jul 24;13(15):3729. doi: 10.3390/cancers13153729 (PMC8345068; doi:10.3390/cancers13153729)
Supplement: Supplementary file 1 [file cancers-13-03729-s001.zip › cancers-1290717-supplementary.pdf]

Supplementary Materials

The Human Fetal and Adult Stem Cell Secretome Can Exert Cardioprotective Paracrine Effects against Cardiotoxicity and Oxidative Stress from Cancer Treatment

Federico Villa, Silvia Bruno, Ambra Costa, Mingchuan Li, Michele Russo, James Cimino, Paola Altieri, Clarissa Ruggeri, Cansu Gorgun, Pierangela De Biasio, Dario Paladini, Domenico Coviello, Rodolfo Quarto, Pietro Ameri, Alessandra Ghigo, Silvia Ravera, Roberta Tasso and Sveva Bollini

Table S1. Biochemical analyses on mNVCM and mNVFib exposed to Dox, with or without hAFS-CM and hMSC-CM priming. Values are expressed as mean ± s.e.m. of at least *n* = 3 independent experiments and refers to data shown in Figure 2 and Figure 3.

Biochemical Analyses	mNVCM						mNVFib					
	**Ctrl**	**Dox**	**hAFS-CM + Dox**	**hMSC-CM + Dox**	**hAFS-CM**	**hMSC-CM**	**Ctrl**	**Dox**	**hAFS-CM + Dox**	**hMSC-CM + Dox**	**hAFS-CM**	**hMSC-CM**
**Oxygen consumption (nmol/min/ 10^6 cells)**	25.75 + 1.39<br>(*n* = 6)	8.51 + 0.62<br>(*n* = 3)	17.8 + 0.43<br>(*n* = 3)	15.03 + 0.66<br>(*n* = 3)	31.84 + 0.52<br>(*n* = 3)	30.5 + 1.42<br>(*n* = 3)	5.28 + 0.15<br>(*n* = 4)	1.1 + 0.15<br>(*n* = 4)	3.99 + 0.24<br>(*n* = 3)	3.35 + 0.21<br>(*n* = 3)	6.16 + 0.41<br>(*n* = 3)	6.29 + 0.22<br>(*n* = 3)
**ATP production (nmol/min/ 10^6 cells)**	62.42 + 2.56<br>(*n* = 6)	14.22 + 1.23<br>(*n* = 3)	42.79 + 0.81<br>(*n* = 3)	36.13 + 0.70<br>(*n* = 3)	78.30 + 1.63<br>(*n* = 3)	74.17 + 2.28<br>(*n* = 3)	12.76 + 0.26<br>(*n* = 4)	1.42 +0.19<br>(*n* = 4)	10.07 + 0.46<br>(*n* = 3)	7.87 + 0.80<br>(*n* = 3)	14.83 + 0.75<br>(*n* = 3)	15.13 + 1.01<br>(*n* = 3)
**OxPhos efficiency (P/O P/M ratio)**	2.43 + 0.09<br>(*n* = 6)	1.67 + 0.04<br>(*n* = 3)	2.40 + 0.03<br>(*n* = 3)	2.41 + 0.13<br>(*n* = 3)	2.46 + 0.06<br>(*n* = 3)	2.43 + 0.63<br>(*n* = 3)	2.41 + 0.03<br>(*n* = 4)	1.32 + 0.20<br>(*n* = 4)	2.52 + 0.14<br>(*n* = 3)	2.34 + 0.09<br>(*n* = 3)	2.41 + 0.04<br>(*n* = 3)	2.41 + 0.07<br>(*n* = 3)
**Glucose consumption (mM /10^6 cells)**	9.05 + 0.17<br>(*n* = 6)	11.45 + 0.21<br>(*n* = 3)	10.06 + 0.15<br>(*n* = 3)	10.05 + 0.20<br>(*n* = 3)	12.53 + 0.14<br>(*n* = 3)	12.36 + 0.30<br>(*n* = 3)	14.80 + 0.49<br>(*n* = 4)	17.74 + 0.49<br>(*n* = 4)	16.65 + 0.40<br>(*n* = 3)	16.49 + 0.38<br>(*n* = 3)	16.97 + 0.56<br>(*n* = 3)	15.78 + 0.23<br>(*n* = 3)
**Lactate release (mM /10^6 cells)**	0.62 + 0.09<br>(*n* = 6)	3.44 + 0.29<br>(*n* = 3)	1.43 + 0.18<br>(*n* = 3)	1.27 + 0.08<br>(*n* = 3)	0.43 + 0.02<br>(*n* = 3)	0.45 + 0.30<br>(*n* = 3)	4.28 + 0.18<br>(*n* = 4)	8.67 + 0.57<br>(*n* = 4)	5.95 + 0.24<br>(*n* = 3)	6.30 + 0.27<br>(*n* = 3)	2.65 + 0.22<br>(*n* = 3)	3.09 + 0.16<br>(*n* = 3)
**% Lactate Fermentation**	3.43 + 0.48<br>(*n* = 6)	15.07 + 1.49<br>(*n* = 3)	6.33 + 0.47<br>(*n* = 3)	7.16 + 0.99<br>(*n* = 3)	1.74 + 0.07<br>(*n* = 3)	1.81 + 0.17<br>(*n* = 3)	14.46 + 0.43<br>(*n* = 4)	24.41 + 0.94<br>(*n* = 4)	17.88 + 1.11<br>(*n* = 3)	19.11 + 1.11<br>(*n* = 3)	7.80 + 0.57<br>(*n* = 3)	9.80 + 0.62<br>(*n* = 3)

Table S2. Left ventricle functional parameters in mice exposed to Dox, with or without hAFS-CM and hMSC-CM priming. Values are expressed as mean ± s.e.m. from echocardiography evaluation and are shown in Figure 5.

Cardiac Function Parameters	Dox (*n* = 6)	hAFS-CM + Dox (*n* = 5)	hMSC-CM + Dox (*n* = 6)	Dox (*n* = 6)	hAFS-CM + Dox (*n* = 5)	hMSC-CM + Dox (*n* = 6)
**Day**	0	0	0	42	42	42
**LVEDd (mm)**	3.6 ± 0.08	3.8 ± 0.03	3.6 ± 0.03	3.7 ± 0.12	4.1 ± 0.09	3.8 ± 0.12
**LVEDs (mm)**	2.3 ± 0.08	2.4 ± 0.03	2.2 ± 0.07	2.7 ± 0.04$^{\#\#}$	2.7 ± 0.14	2.5 ± 0.11
**IVSd (mm)**	0.50 ± 0.01	0.53 ± 0.03	0.51 ± 0.03	0.50 ± 0.02	0.60 ± 0.02	0.54 ± 0.02
**IVSs (mm)**	0.90 ± 0.02	1.0 ± 0.03	0.92 ± 0.03	0.85 ± 0.02	0.99 ± 0.04	0.97 ± 0.05
**LVPWd (mm)**	0.50 ± 0.03	0.55 ± 0.03	0.51 ± 0.03	0.46 ± 0.02	0.55 ± 0.03	0.54 ± 0.02
**LVPWs (mm)**	0.89 ± 0.05	0.94 ± 0.05	0.84 ± 0.04	0.68 ± 0.06	0.87 ± 0.07	0.83 ± 0.05
**FS (%)**	37.0 ± 1.3	37.8 ± 1.0	37.5 ± 1.4	26.8 ± 1.3$^{\#\#\#\#}$	34.0 ± 2.2**	34.3 ± 1.2**
**EF (%)**	66.1 ± 2.1	68.6 ± 1.3	68.4 ± 1.8	52.1 ± 2.8$^{\#\#\#}$	63.1 ± 3.0	63.9 ± 1.6*

Dox day 0 vs day 42: $^{\#\#}$ $p < 0.01$, $^{\#\#\#}$ $p < 0.001$, $^{\#\#\#\#}$ $p < 0.0001$; hAFS-CM + Dox and hMSC-CM +Dox vs Dox at day 42: * $p < 0.05$, ** $p < 0.01$ by two-way repeated-measures ANOVA with Tukey's post-hoc test. LVEDd: left ventricular end-diastolic diameter; LVEDs: left ventricular end-systolic diameter; IVSd: interventricular septal thickness at end-diastole; IVSs: interventricular septal thickness at end-systole; LVPWd: left ventricular posterior wall thickness at end-diastole: LVPWs, left ventricular posterior wall thickness at end-systole; FS: fractional shortening; EF: ejection fraction; mm: millimeter.

Table S3. Biochemical analyses on hearts of mice treated with Dox after hAFS-CM and hMSC-CM priming. Values are expressed as mean ± s.e.m. of at least *n* = 3 independent experiments and refer to data shown in Figure 5.

Biochemical Analyses	Ctrl	Dox	hAFS-CM + Dox	hMSC-CM + Dox
**Glucose-6 phosphate dehydrogenase (G6PD) activity (mU/mg)**	7.66 ± 0.11 (*n* = 3)	11.59 ± 0.12 (*n* = 4)	8.68 ± 0.05 (*n* = 5)	8.88 ± 0.09 (*n* = 3)
**Glutathione reductase (GRx) activity (mU/mg)**	66.54 ± 2.74 (*n* = 3)	36.90 ± 0.51 (*n* = 4)	44.74 ± 1.44 (*n* = 5)	46.46 ± 0.51 (*n* = 3)
**Catalase activity (mU/mg)**	153.84 ± 13.37 (*n* = 3)	86.66 ± 3.64 (*n* = 4)	124.49 ± 10.79 (*n* = 5)	127.48 ± 6.48 (*n* = 3)
**Malondialdheyde (MDA) intracellular concentration (µM MDA/mg)**	10.39 ± 0.21 (*n* = 3)	42.30 ± 0.42 (*n* = 4)	25.17 ± 0.37 (*n* = 5)	25.28 ± 0.58 (*n* = 3)
**Complex I (MRC I) activity (mU/mg)**	1.1 ± 0.03 (*n* = 3)	0.44 ± 0.02 (*n* = 4)	0.76 ± 0.02 (*n* = 5)	0.73 ± 0.02 (*n* = 3)

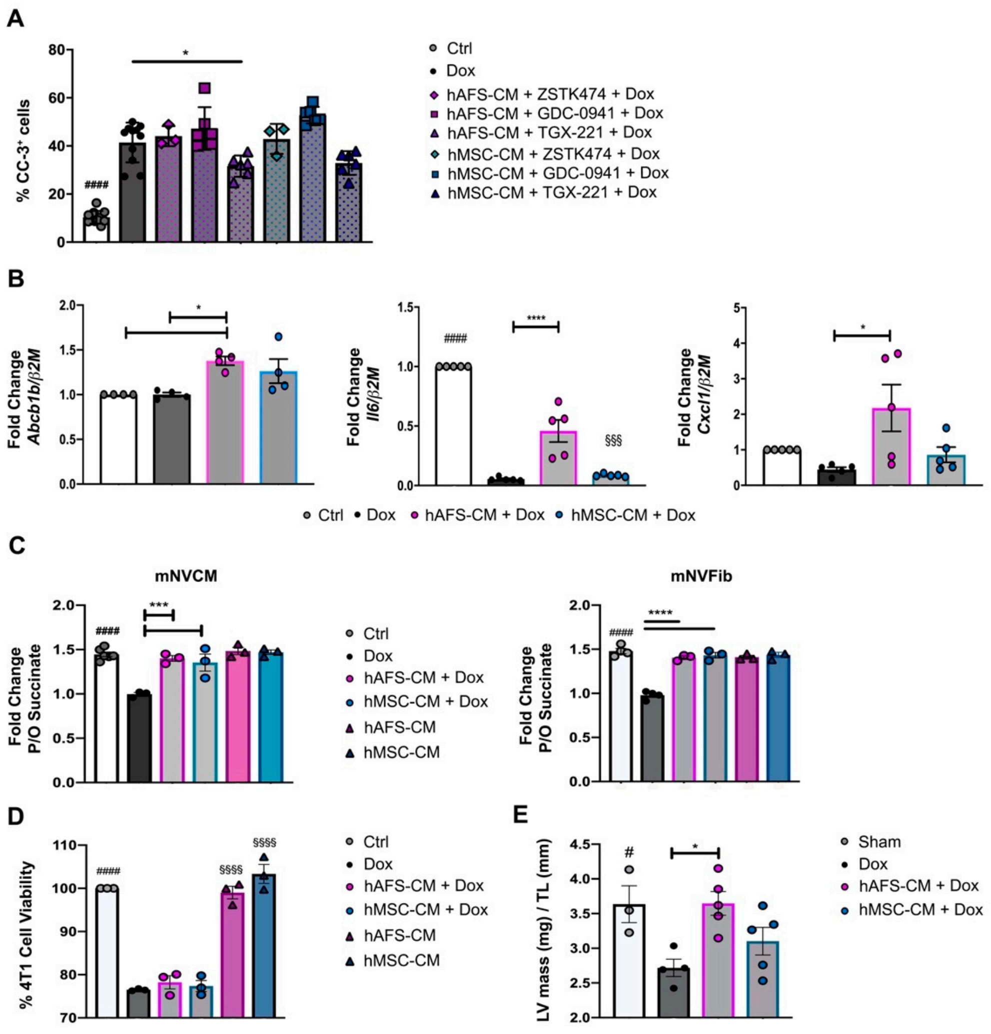

Figure S1. A. Percentage of mNVCM expressing cleaved-caspase-3 (*%CC-3⁺ cells*) in control condition (n = 11, *Ctrl: 10.35 ± 0.80%*), after exposure to 1 µM Dox with or without (n = 11, *Dox: 41.43 ± 2.50%*) pre-incubation with hAFS-CM or hMSC-CM in combination with the pan class I PI3K inhibitor ZSTK474, the PI3Kα and PI3Kδ inhibitor GDC-0941 and the PI3Kβ inhibitor TGX-221 (n = 3, *hAFS-CM + ZSTK474 + Dox: 44.06 ± 2.39%*; n = 6, *hAFS-CM + GDC-0941 + Dox: 47.24±3.63%*; n = 6, *hAFS-CM + TGX-221 + Dox: 31.60 ± 1.80%*; n = 3, *hMSC-CM + ZSTK474 + Dox: 42.93 ± 3.58%*; n = 6, *hMSC-CM + GDC-0941 + Dox: 52.78 ± 1.40%*; n = 6, *hMSC-CM + TGX-221 + Dox: 32.90 ± 2.00%*). Values are expressed as mean ± s.e.m. of independent experiments; *Ctrl* vs. *Dox* ####p < 0.0001; *hAFS-CM + TGX-221 + Dox* vs. *Dox* * p = 0.0393. **B.** Real time qRT-PCR analysis on mNVCM after exposure to 1 µM Dox with or without (*Dox*) pre-incubation with hAFS-CM (*hAFS-CM + Dox*) or hMSC-CM (*hMSC-CM+ Dox*) versus mNVCM in control condition (*Ctrl,* considered as calibrator*)* for the fold-change expression of *Abcb1b* (n = 4, *Dox: 1.00±0.02; hAFS-CM + Dox: 1.40± 0.05; hMSC-CM + Dox: 1.26 ± 0.13; hAFS-CM + Dox* vs. *Dox and hAFS-CM + Dox* vs. *Ctrl, *p = 0.0146); Il6* (n = 5, *Dox: 0.05±0.01; hAFS-CM + Dox: 0.46 ± 0.10; hMSC-CM + Dox: 0.08±0.005; Ctrl* vs. *Dox, ####p* < 0.0001*; hAFS-CM + Dox* vs *Dox, ****p* < 0.0001 *and hAFS-CM + Dox vs. hMSC-CM + Dox,* §§§p = 0.0002*)* and *Cxcl1* (n = 5, *Dox: 0.44 ± 0.07; hAFS-CM + Dox: 2.17 ± 0.66; hMSC-CM + Dox: 0.86 ± 0.21; hAFS-CM + Dox* vs. *Dox, *p* < 0.0136*)* over the housekeeping gene *Beta-2 Microglobulin* (*β2M*). **C.** From the top left corner: mNVCM oxygen consumption rate stimulated with succinate in control condition (n = 6, *Ctrl: 18.91± 0.95*), after exposure to 1 µM Dox (n = 3, *Dox: 4.98± 0.58),* pretreated with hAFS-CM (n = 3 *hAFS-CM +Dox: 13.06 ± 0.86*) or hMSC-CM (n = 3, *hMSC-CM + Dox: 11.73± 1.05*) before the Dox stimulation, or only pretreated with hAFS-CM (n= 3, *hAFS-CM: 27.42 ± 0.60*) or hMSC-CM (n = 3, *hMSC-CM: 26.10± 0.90*). mNVFib oxygen consumption rate stimulated with succinate in control condition (n= 4, *Ctrl: 4.25±0.17*), after exposure to 1 µM Dox (n = 4, *Dox: 0.98 ± 0.10),* pretreated with hAFS-CM (n = 3 *hAFS-CM +Dox: 3.74±0.25*) or hMSC-CM (n = 3, *hMSC-CM + Dox: 3.06 ± 0.19*) before the Dox stimulation, or only pretreated with hAFS-CM (n= 3, *hAFS-CM: 5.44± 0.35*) or hMSC-CM (n = 3, *hMSC-CM: 5.45 ± 0.46*). In each panel, values are expressed as mean ± s.e.m. of independent experiments; *Ctrl* vs. *Dox* ####p < 0.0001; *hAFS-CM + Dox* vs. *Dox and hMSC-CM +Dox* vs. *Dox* ***p < 0.001 (mNVCM, p = 0.0002 and p = 0.0008, respectively) or ****p < 0.0001 (mNVFib). **D.** 4T1 cell viability percentage by MTT assay normalized to untreated control cells (n = 3, *Ctrl*) considered as 100% viable, after exposure to 1 µM Dox with or without (n = 3, *Dox: 76.51 ± 0.15%*) pre-incubation with hAFS-CM (n = 3, *hAFS-CM + Dox: 78.25±1.53%*) or hMSC-CM (n = 3, *hMSC-CM + Dox*: *77.37 ± 1.30%*) or tretaed with hAFS-CM (n = 3, *hAFS-CM*: *99.03±1.44%*) or hMSC-CM (n = 3, *hMSC-*

CM + Dox: *103.35 ± 2.20%*) only. Values are expressed as mean ± s.e.m. of independent experiments; *Ctrl* vs. *Dox* $^{####}$ $p < 0.0001$; *hAFS-CM* vs. *Dox* and *hMSC-CM* vs. *Dox* §§§§$p < 0.0001$. **E.** Left ventricle (LV) mass (milligram, *mg*) over tibial length (TL, in millimeter, *mm*) ratio in sham healthy mice ($n = 3$, *Sham: 3.64 ± 0.27*) treated with a cumulative dose of 12 mg/Kg Dox ($n = 4$, *Dox: 2.72 ± 0.18)* with or without either hAFS-CM ($n = 5$, *hAFS-CM + Dox: 3.65 ± 0.11)* or hMSC-CM ($n = 5$, *hAFS-CM + Dox: 3.10 ± 0.20)* after 42 days. Values are expressed as mean ± s.e.m. of independent animals; *Sham* vs. *Dox* $^{\#}p = 0.0383$; *hAFS-CM + Dox* vs. *Dox* * $p = 0.0163$.
